# Enhancing Machine Learning Performance for ALS Classification Using Feature Selection Technique

**DOI:** 10.3390/diagnostics16213573

**Published:** 2026-10-29

**Authors:** Arunesh Dutt, Ashutosh Tiwari, Saurav Tripathi, Pradeep Kumar Tiwari, Mritunjai Kumar, Akansha Dwivedi, Komal Singh, Ashish Khare, Narendra Kumar Shukla, Niraj Kumar

**Affiliations:** 1 Department of Electronics and Communication Engineering, University of Allahabad, Prayagraj 211002, India; aruneshdutt19@gmail.com (A.D.); khare@allduniv.ac.in (A.K.);; 2 Department of Neurology, All India Institute of Medical Sciences (AIIMS), Rishikesh 249203, Indiamritunjaisingh68@gmail.com (M.K.); 3 Department of Neurology, All India Institute of Medical Sciences (AIIMS), Gorakhpur 273008, India; 4 Department of Computer Science and Engineering, SRM University, Kattankulathur 603203, India; 5 Centre of Computer Education & Training, Institute of Professional Studies, University of Allahabad, Prayagraj 211002, India; 6 Department of Psychology, Kamla Rai College, Jai Prakash University, Gopalganj 841301, India; 7 Department of Neurology, All India Institute of Medical Sciences (AIIMS), Bibinagar 508126, India

**Keywords:** motor neuron disease (MND), amyotrophic lateral sclerosis (ALS), electromyography (EMG), muscles, machine learning (ML)

## Abstract

**Background**: Electromyogram (EMG) is considered for the diagnosis of amyotrophic lateral sclerosis (ALS). We investigated how frequently an initial electrophysiological evaluation is performed in ALS patients. **Objective**: We aimed to identify ALS cases using the feature selection algorithm (FSA). A pattern of feature involvement was expected in the early phase of ALS based on the typical consistent involvement of a few particular muscles in the late stages. **Methods**: We used a clinical and EMG dataset of 36 patients (21 ALS patients and 15 healthy) for analysis. The present approach used a Random Forest (RF)-based machine learning (ML) algorithm for the prediction of ALS patients. The goal of this study was to determine how feature selection techniques can enhance machine learning algorithms that can be used in real-time applications. **Findings**: The experimental findings demonstrate the superior performance of the proposed model. This work demonstrates how machine learning models can offer important predictive insights in ALS, improving knowledge of disease dynamics and aiding patient care. According to the comparison results, the RF model utilizing the feature selection algorithm demonstrated a positive improvement, and the accuracy of the RF model with and without the feature selection technique was found to be 87.50% and 75%, respectively. **Conclusions**: The algorithm improved the model accuracy from 75% to 87.50%, resulting in an absolute increase of 12.5 percentage points in classification accuracy. This study found that fibrillation, positive sharp waves (PSW), and fasciculation features were high-yielding in EMG of ALS cases.

## 1. Introduction

For more than 100 years, the pathophysiology of amyotrophic lateral sclerosis (ALS) has been a mystery. To find a medication that can delay or stop the progression of ALS, various therapeutic studies have recently been conducted. It appears that everyone agrees that an early course of treatment has a better probability of being somewhat effective [1]. The only practical method to assist the neurologist in making a trustworthy early diagnosis is an appropriate electromyogram (EMG) [2]. The El Escorial criteria were evaluated by specialists in this field at the Airlie House meeting in April 1998 in order to support an earlier diagnosis [3]. Early in the disease, a needle examination might not reveal widespread denervation [2]. Nonetheless, the majority of neurophysiologists rejected the EMG diagnosis of ALS unless at least one muscle showed signs of fibrillation or positive sharp waves (PSW) [4]. Fasciculations are tiny, haphazard muscular movements that can happen in both healthy individuals and a variety of peripheral nervous system illnesses [5,6,7]. Widespread fasciculations, on the other hand, are a common clinical characteristic of ALS and are thought to be strongly indicative of ALS. The inclusion of fasciculations in the Awaji criteria for amyotrophic lateral sclerosis (ALS) highlights their clinical significance [8]. We believed that, for the most part, we had seen ALS patients with fibs, positive sharp waves, and fasciculations earlier in the course of the illness. Numerous classification and prediction techniques [9,10,11,12,13] have been employed to forecast how ALS patients’ health state will develop. However, prognostic models frequently lack precision and may not always give information on the uncertainty and interpretability of outcomes, which are critical for physicians because patients are so diverse. This problem is rarely addressed. For a number of reasons, especially in fields like healthcare, machine learning models’ interpretability and complexity are crucial. Firstly, it makes it possible for medical professionals to comprehend and trust the model’s forecasts. A doctor could be reluctant to make judgments based on a model’s prediction of a particular prognosis if it is unable to provide an explanation. Additionally, interpretability is crucial for model validation and verification. Interpretability is necessary for transparency and accountability. Although machine-learning techniques have increasingly been investigated for EMG-based ALS analysis, to the best of our knowledge, no previous study identified in our literature review has specifically combined machine learning with wrapper-based feature selection for ALS classification using the particular EMG characteristics considered in the present study. These are fibrillation potentials, positive sharp waves (PSWs), and fasciculations. The present study therefore investigates whether a compact subset of clinically relevant EMG characteristics can provide useful discrimination between ALS and healthy subjects. The proposed framework focuses on identifying informative EMG characteristics while reducing the dimensionality of the input data, thereby providing a potentially more interpretable and computationally simpler classification framework.

### Contributions

This paper highlights the presence and influence of machine learning algorithms using an EMG dataset. Here, we used a supervised ML technique to develop an accurate prediction model. Our approach mainly uses sophisticated feature selection methods that minimize the models’ complexity while improving their forecast accuracy and interpretability. Here are our particular contributions:

- We draw attention to how redundant and/or unnecessary features can reduce machine learning algorithms’ accuracy. By eliminating unnecessary characteristics, we demonstrate how feature selection can enhance ALS disease prediction and classification models.
- By analyzing the features that most influence model choices, we show how feature selection can result in domain insights.
- Using the RF-based framework, we were able to enhance the machine learning algorithm performance on actual EMG data.

The remaining work is organized as follows: Methodology is provided in Section 2. Section 3 displays the results, and Section 4 presents the discussion of the findings. Section 5 contains the conclusion and plans for future work.

## 2. Materials and Methods

### 2.1. Study Design and Participants

This cross-sectional study was carried out between 1 September 2023 and 14 October 2024. Electrophysiological testing and data collection were carried out at a university hospital with tertiary care. The Engineering Institute lab was mostly used for data analysis and algorithm development. Following a Memorandum of Understanding between the two participating institutions, the Engineering Institute and a tertiary care university hospital, the institutional ethics committee of both institutions approved the protocol (Ethics approval no. AIIMS/IEC/23/196). After acquiring written informed consent from patients or their caregivers, participants were enrolled.

The information in this database is often utilized to assess the efficacy of novel ALS treatments as well as to gain a deeper understanding of the disease’s characteristics and the related risk variables. Because not all patients were followed for the same amount of time or at regular intervals, and because assessments took place at different times depending on the patient, variability in data collection was expected.

**Description of dataset:** The dataset used in this study comprised 36 subjects, including 21 patients with amyotrophic lateral sclerosis (ALS) and 15 healthy participants. The decision (target) attribute was the diagnostic class of each subject, with two classes: ALS and Healthy. Thus, the classification task was to distinguish ALS patients from healthy participants using the electromyographic features described in Table 1. For the classification analysis, the two classes were coded as Class 0 = ALS and Class 1 = Healthy, consistent with the coding used in the confusion matrices. The distribution of subjects was therefore 21 in the ALS class and 15 in the Healthy class. The dataset included 21 ALS subjects and 15 healthy subjects, resulting in a mildly imbalanced class distribution. This class imbalance was considered when interpreting the model performance, particularly for accuracy, sensitivity, and specificity. Given the small sample size, the reported performance estimates should be interpreted cautiously. In this section, we present types of experimental datasets and some clinical measurements as follows:

**(i)**  **Clinical Data Measurement**

The ALS patients’ clinical and demographic data were gathered. Every patient had a thorough clinical evaluation. The ALS Functional Rating Scale-Revised (ALSFRS-R) [14], Modified Rankin Scale (mRS) [15], and Functional Gait Assessment (Gait) [16] were used to measure disability. Cognitive functioning was evaluated using the Mini-Mental State Examination (MMSE) [17] and MoCA [18].

**(ii)**  **Electromyogram (EMG) Data Measurement**

Every patient was evaluated using an EMG test according to the Awaji-Shima criteria and a clinical diagnosis of ALS [19]. Concentric needle electrodes were used to record the EMG parameters. The head and trunk, upper extremity, and lower extremity were the three phases of the EMG evaluation in this case.

Although all electrophysiological examinations were conducted in the same university hospital setting, variability was observed in the EMG characteristics among participants. This variability may be related to differences in individual clinical presentation, disease duration, severity, muscle involvement, and electrophysiological response. Consequently, features such as spontaneous activity, recruitment pattern, and motor unit characteristics may differ across participants. Such inter-subject variability is inherent to clinical EMG data and was retained in the dataset to reflect the characteristics of the study population. However, this variability may influence classification performance and should be considered when interpreting the generalizability of the proposed model.

The presence of missing or unavailable values for some EMG features should not be interpreted as evidence that different EMG acquisition protocols were used. The electrophysiological examinations were conducted in the same university hospital setting. Variability in the recorded EMG features may instead reflect differences in the clinical and electrophysiological characteristics of individual participants, as not all spontaneous or motor-unit abnormalities are necessarily observed in every subject. Therefore, the availability of a particular EMG feature reflects the findings obtained during the individual examination rather than a change in the acquisition protocol.

**(iii)**  **Disease Validation**

Expert investigations and clinical diagnoses were taken into consideration. AT and MK, two neurologists, verified the disease’s clinical diagnosis. To determine the diagnosis and early prediction, an ML model was used on this dataset. The purpose of this study was to use a minimum and the selection of relevant features in machine learning for ALS prediction using a feature selection technique.

**(iv)**  **Feature Selection**

Some EMG features were unavailable for certain participants. Missing data can affect the reliability of machine learning models and may introduce selection bias, particularly when the pattern of missingness is not completely random. In the present study, participants with missing values in the required EMG features were excluded from the final analysis to ensure a consistent set of input variables for model development. No imputation or Principal Component Analysis (PCA) was performed in the final analysis. We acknowledge that complete-case exclusion may reduce the effective sample size and may introduce selection bias; therefore, this limitation should be considered when interpreting the findings. The quantity and quality of data are particularly important in medical machine learning, where heterogeneous clinical datasets can affect model development and generalizability [20]. Furthermore, the complexity of the feature selection problem increases with the number of input features [21,22].

Feature selection refers to the process of identifying the most relevant features from a dataset while removing irrelevant or redundant variables. It is an important step in machine learning, particularly when dealing with datasets containing multiple input features. By reducing data dimensionality, feature selection can improve model interpretability, reduce computational complexity, and potentially enhance predictive performance [23,24,25]. The primary objective of feature selection is to enable machine learning models to focus on the most informative variables, thereby improving generalization and prediction accuracy. It can also reduce model complexity and potentially reduce the risk of overfitting, resulting in models that are easier to interpret and deploy.

Feature selection techniques are generally categorized into three major approaches: filter, wrapper, and embedded methods. Each approach uses a different strategy for evaluating the relevance of features and has its own advantages and limitations [26]. In the present study, a wrapper-based feature selection approach was employed. Wrapper methods evaluate different subsets of features using a predefined machine learning algorithm and identify the feature subset that provides the best classification performance. Common wrapper-based techniques include recursive feature elimination (RFE), forward selection, and backward elimination [27,28,29,30]. Although wrapper methods can identify task-specific feature subsets and potentially improve predictive performance, they may require greater computational resources because multiple feature subsets must be evaluated using the learning algorithm. Therefore, their computational cost may increase with the number of features.

In this study, a wrapper-based feature-selection approach was employed to identify a compact subset of EMG characteristics relevant to ALS classification. Specifically, recursive feature elimination (RFE) was used as a backward elimination strategy. In this approach, all candidate features were initially included in the model and ranked according to their importance. The least important feature or features were then recursively removed, and the model was retrained at each iteration. The performance of each reduced feature subset was evaluated using classification accuracy as the predefined performance criterion. This recursive process continued until the desired number of features was reached. Based on the evaluation results, the final feature subset consisted of three EMG characteristics: fibrillation potentials, positive sharp waves (PSWs), and fasciculations. These features were subsequently used as inputs to the RF model for ALS classification.

**(v)**  **System Design Specification**

The entire study is conducted using Python 3.12.3, and the libraries used are pandas, numpy, matplotlib, seaborn, scikit-learn, openpyxl, and xlrd. The key hyperparameter values were ‘n_estimators’ [50, 100], ‘max_depth’ [1, 2, 3], ‘min_samples_split’ [4, 6, 8], ‘max_features’ [“sqrt”], and ‘random_state’ [42].

**(vi)**  **Model Evaluation Criteria**

Evaluation is essential for determining the performance and dependability of an ML model. It helps us compare several methods to determine which is optimal for a given task and lets us know how effectively the model generalizes to new, unknown data. Overfitting, in which a model performs well on training data but badly on new data, is a danger associated with improper evaluation. In order to refine and fine-tune the model for better outcomes, evaluation also helps discover any biases and inaccuracies. These metrics are derived using Python’s scikit-learn metrics. Evaluation aids in determining the model’s advantages and disadvantages, directing future developments, and guaranteeing that ML models are appropriate for practical uses. We calculated the number of true positives (TP), false negatives (FN), true negatives (TN), and false positives (FP) to evaluate the predictive quality of our classification model. These criteria led to the derivation of metrics like sensitivity, specificity, accuracy, precision, F1-score, and AUC for both classes. The model information is provided by the matrix, which helps with performance assessment and improvement.

**(vii)**  **Proposed Model building process**

ALS classification was performed using a Random Forest (RF)-based machine learning approach. The performance of the proposed model was evaluated using several performance metrics, including sensitivity, specificity, accuracy, precision, F1-score, and area under the receiver operating characteristic curve (AUC) [31,32]. Nested cross-validation was applied to the training data to assess model performance [33], while the confusion matrix was used to derive the classification metrics. The model classified the subjects into two groups: ALS and healthy. Following preprocessing, the dataset comprised 36 subjects, including 21 ALS patients and 15 healthy participants. The dataset was divided into training and independent testing subsets. The training subset was used for RF model development, and nested cross-validation was performed within the training data to evaluate the model during development.

**Figure 1:**
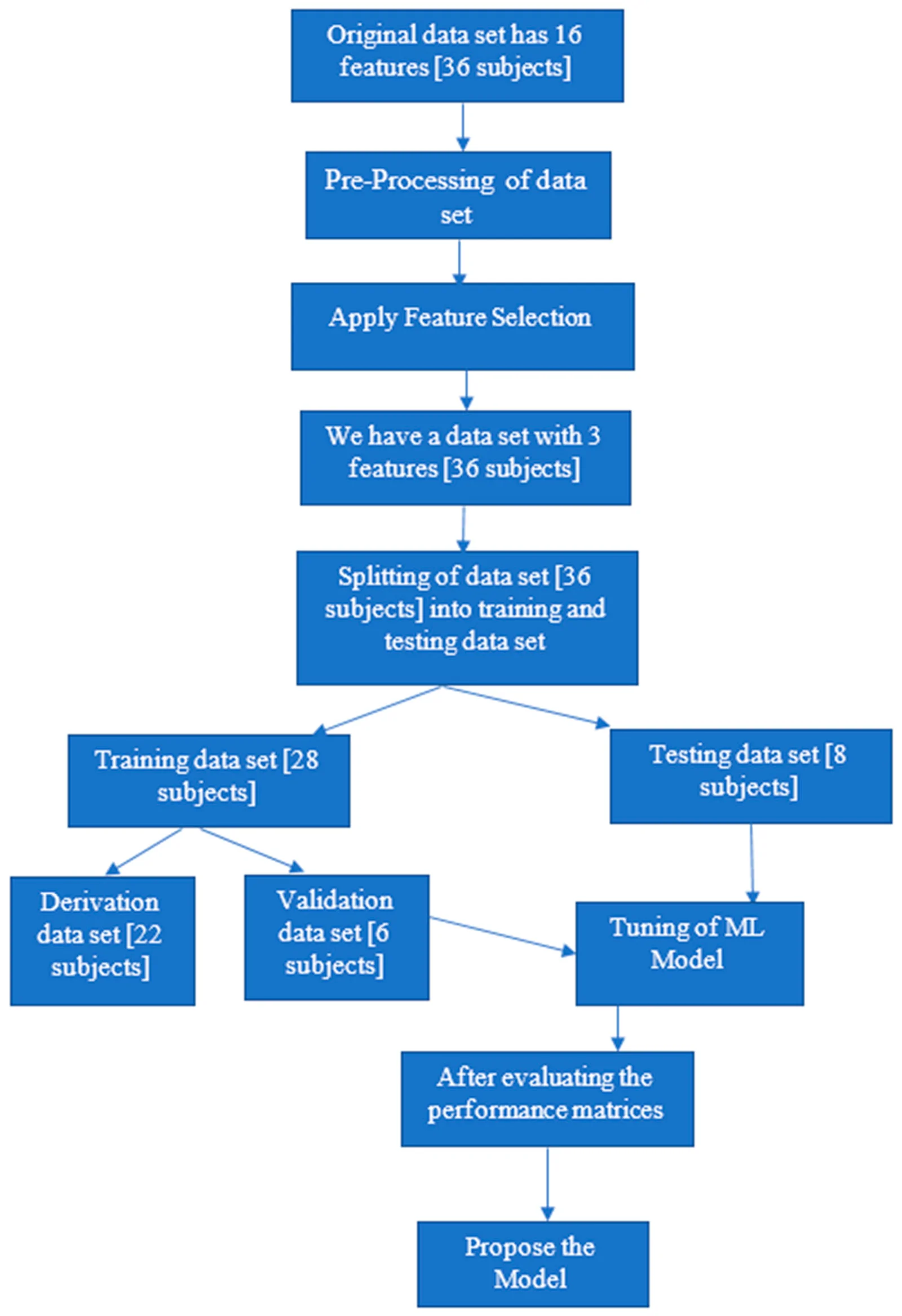
Prediction model of ALS.

To minimize the risk of information leakage during feature selection and model evaluation, a nested cross-validation framework was employed on the model-development dataset. The outer cross-validation was used to estimate the generalization performance of the classification model, while an inner cross-validation procedure was used for wrapper-based feature selection and model/hyperparameter optimization. For each outer fold, the feature-selection procedure was performed exclusively using the training portion of that fold. The selected feature subset and optimized model were then applied to the corresponding outer validation fold, which remained unseen during feature selection and model optimization. This process was repeated for all outer folds, ensuring that information from the validation data did not influence the feature-selection process. After completion of the nested cross-validation procedure, the finalized model was trained using the model-development data according to the established feature-selection and model-development procedure. The independent test set was kept completely unseen throughout feature selection, hyperparameter optimization, and cross-validation and was used only for the final evaluation of the finalized model. Figure 1 illustrates the proposed RF-based machine learning framework for ALS classification using EMG features.

### 2.2. Input Features and Feature Description

The initial dataset consisted of 16 demographic and electromyographic features. The complete list of input variables, their definitions, and coding schemes is presented in Table 1.

## 3. Results

Following preprocessing, 28 subjects were used for model development, and eight subjects were reserved as an independent test set. Nested cross-validation was performed within the training subset to assess model performance during development. The final performance metrics were subsequently evaluated on the independent test set. Figure 1 shows the proposed model framework for ALS prediction. ALS patients in the study cohort had an average age of 44.95 years, with a range of 24 to 58. The mean ALSFRS-R of the study participants was 29.24 (ALSFRS-R was less than 24 in five ALS patients), and the mean mRS was 2.5 in three ALS patients with mRS > 5 (severe impairment). Ten ALS patients had a mean gait score of 17.62, which was less than 24. The mean MMSE was 23.24 (MMSE < 24 in one ALS patient), and the mean MoCA was 23.33 (MoCA < 24 in two ALS patients). Healthy individuals ranged in age from 22 to 51, with a mean age of 37.93. Out of 21 ALS patients, 10 patients had definite ALS at the time of the investigation, four had probable ALS, and four had possible ALS. Three ALS patients had the need for a feeding tube and/or non-invasive respiratory assistance (NIV). Routine blood tests and relevant radiological investigations were performed on all patients in order to rule out other diseases.

Table 2 shows the clinical and demographic information and maximum values of all the scores used in our study. The experiments carried out and the performance metrics chosen to evaluate the model efficacy are explained in depth in this section. The experiment findings are then reported together with comparisons to pertinent earlier research using various characteristics associated with ALS. The dataset characteristics are reported in Table 2, consisting of ALS and healthy individuals.

**Figure 2:**
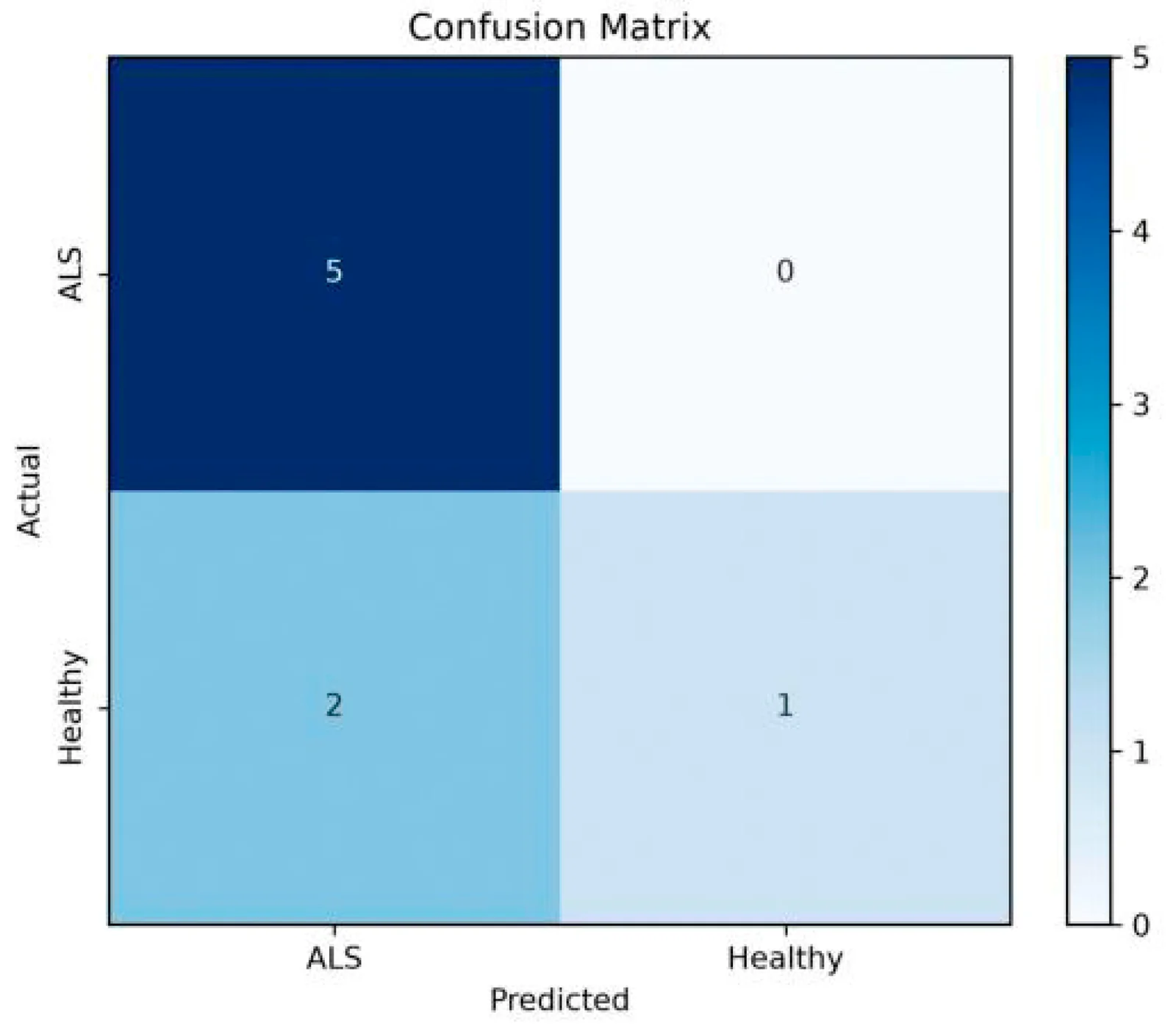
Confusion matrix of the proposed RF-based ML model without FSA.

**Figure 3:**
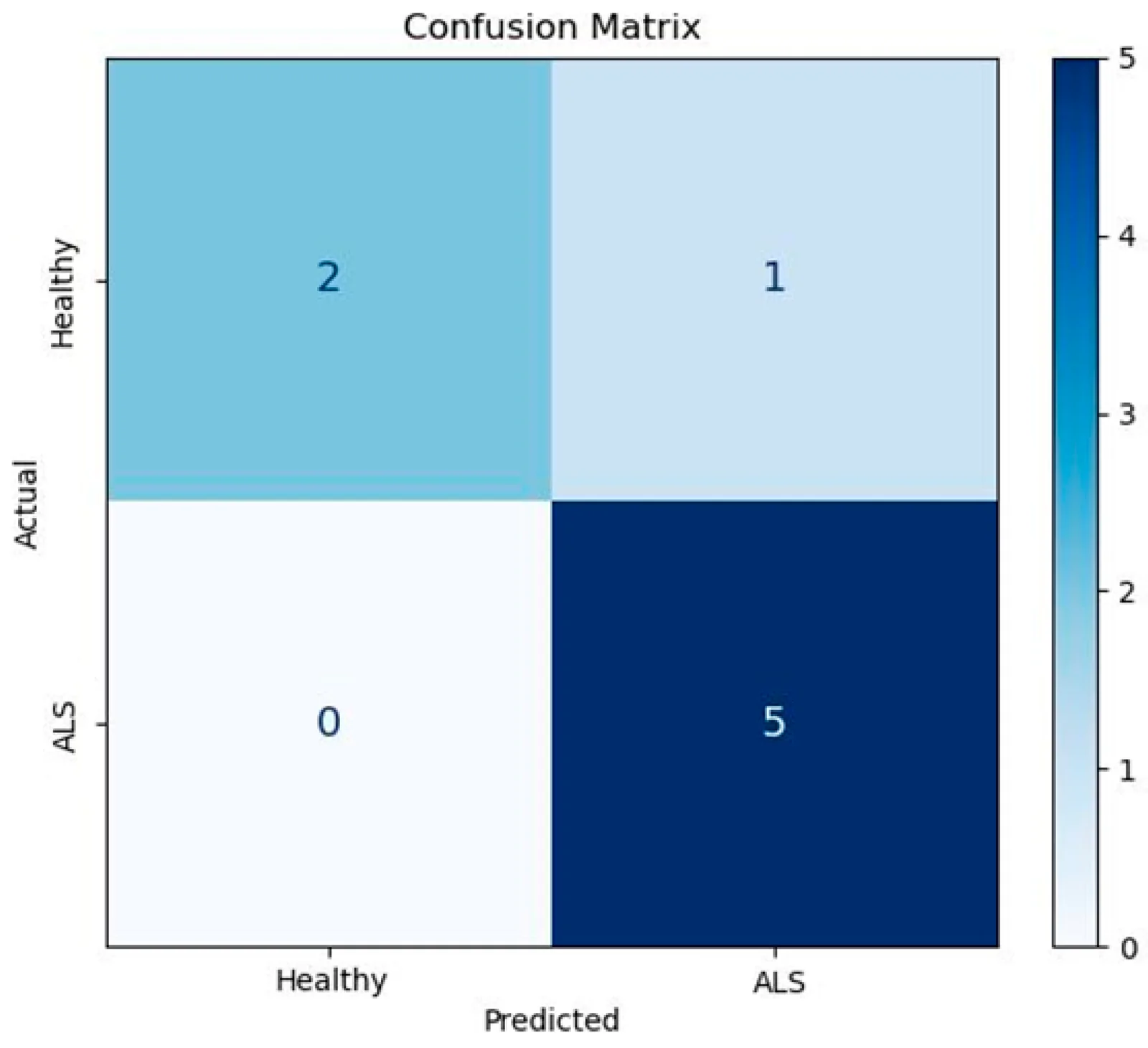
Confusion matrix of the proposed RF-based ML model with FSA.

The performance metrics reported in Table 3 and Table 4 were calculated from the corresponding confusion matrices obtained from the Random Forest (RF)-based model, shown in Figure 2 and Figure 3, respectively. The confusion matrices provide the numbers of true positives (TP), true negatives (TN), false positives (FP), and false negatives (FN) based on the model predictions and actual class labels. Sensitivity was calculated as TP/(TP + FN), specificity as TN/(TN + FP), accuracy as (TP + TN)/(TP + TN + FP + FN), precision as TP/(TP + FP), and F1-score as 2*Precision*Recall/(Precision + Recall). The AUC was obtained from the corresponding receiver operating characteristic (ROC) analysis. Thus, Table 3 and Table 4 summarize the performance metrics derived from the underlying classification results for both classes, while Figure 2 and Figure 3 provide the corresponding confusion matrices used for these calculations. The analysis was performed for the RF model without feature selection (FSA) and with FSA using the same study cohort of eight participants.

The Random Forest (RF) model achieved an accuracy of 75.00% without feature selection (FSA) and 87.50% after applying FSA. This represents an observed absolute increase of 12.5 percentage points in classification accuracy. The observed change in accuracy corresponds to only a small number of additional correctly classified subjects and therefore should not be interpreted as evidence of a statistically significant improvement by accuracy comparison alone.

To provide a more appropriate interpretation of model performance, the uncertainty associated with the accuracy estimates should be considered. Therefore, the reported accuracy values are presented as descriptive performance measures rather than as evidence of statistically significant superiority of the FSA-based model. Although the FSA-based RF model showed an observed improvement in accuracy compared with the model without FSA, the limited sample size reduces the statistical power of the comparison and may affect the generalizability of the findings.

A formal paired comparison, such as McNemar’s test, would require subject-level paired predictions from both models evaluated on exactly the same observations. Since the present analysis primarily reports aggregate model performance obtained during cross-validation, a formal paired significance test was not used to claim statistical superiority. Accordingly, the improvement from 75.00% to 87.50% is interpreted as an observed performance gain that requires confirmation using a larger independent dataset and subject-level paired predictions.

The performance of the proposed Random Forest (RF) model was evaluated using confusion matrices derived from the independent test data. In the present analysis, ALS was defined as the positive class (Class 0), whereas Healthy was defined as the negative class (Class 1). Accordingly, sensitivity represents the proportion of ALS subjects correctly classified as ALS, while specificity represents the proportion of healthy subjects correctly classified as healthy. Accuracy represents the proportion of subjects correctly classified by the model. Precision represents the proportion how many were actually positive by the model. F1-score represents how well the model balances precision and recall. AUC generally distinguishes the classes, and a higher AUC means better discrimination. The confusion matrices for the RF model without feature selection (FSA) and with FSA are presented in Figure 2 and Figure 3, respectively, and the corresponding performance measures are reported in Table 3 and Table 4. The confusion matrices and performance measures were recalculated using both class encodings for both models to ensure consistency among the reported sensitivity, specificity, accuracy, precision, F1-score, and AUC values.

The RF model was evaluated using the original set of 16 features and the feature-selection set comprising three selected features: fibrillation, positive Sharp waves (PSW), and fasciculation. Thus, the feature-selection procedure reduced the input feature set from 16 to three features while retaining the most informative EMG characteristics identified by the wrapper-based feature-selection approach. The resulting sensitivity, specificity, accuracy, precision, F1-score, and AUC values for both without and with FSA are presented in Table 3 and Table 4. The comparison was performed to assess the effect of feature reduction on the classification performance of the RF model. All performance measures were calculated from the corresponding confusion matrices using the same class definitions, with ALS consistently treated as the positive class and healthy as the negative class.

Some earlier work has been carried out using statistical analysis methods, shown in Table 5. In this work, most of the studies used a number of features of one, and only a single study shows three features.

**Figure 4:**
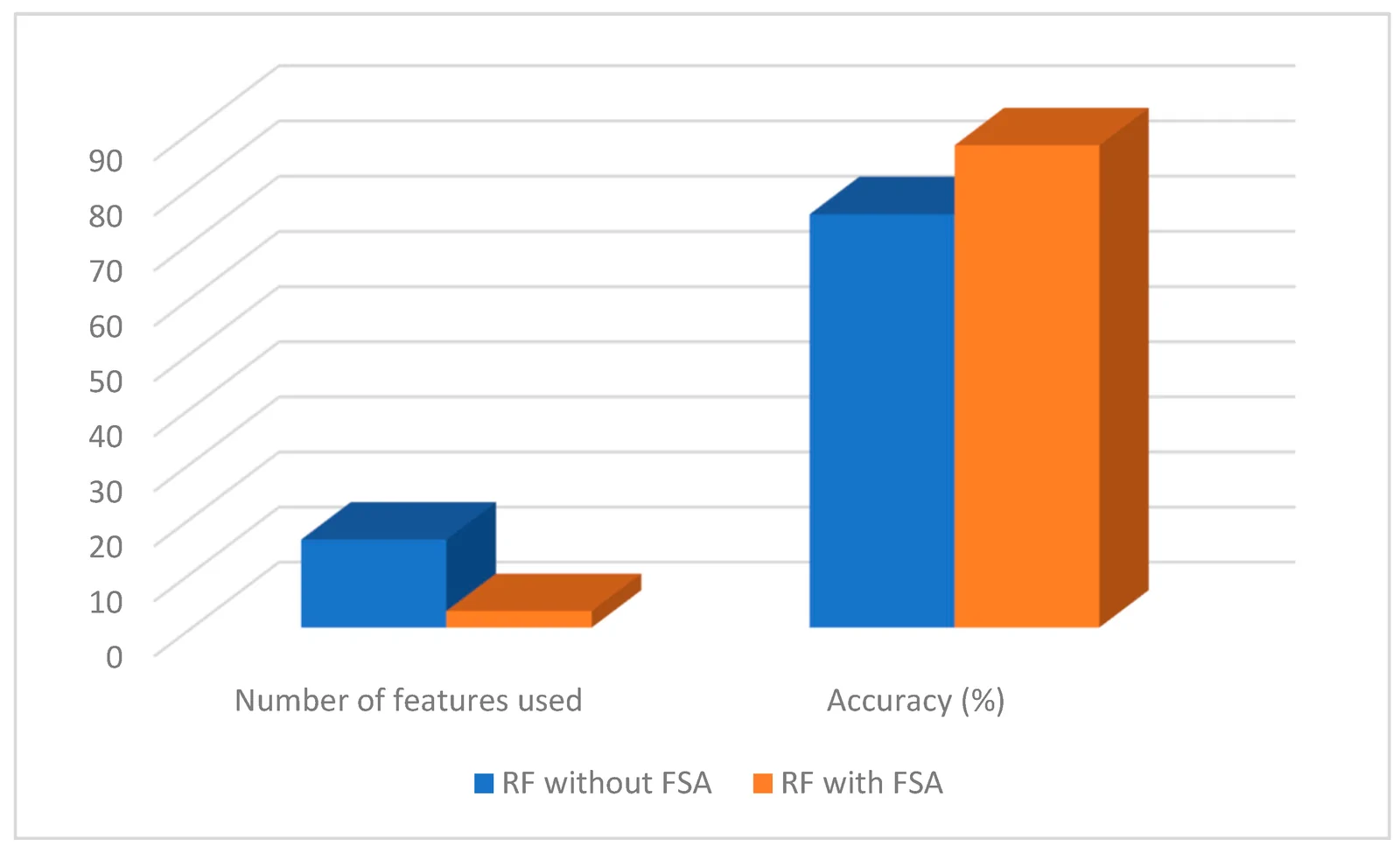
Comparison of number of features and accuracy without and with FSA.

The accuracy of the model predictions and its ability to differentiate between ALS and healthy classes are assessed using this matrix. By comparing the model predictions with the dataset’s actual labels, it makes it easier to compute a variety of assessment measures and offers a straightforward evaluation process. Here in this work, the number of features used without FSA data was 16; after using FSA data, the number of features was reduced to three. The accuracy of the proposed ML model implemented in this research is compared in Figure 4. It can be seen that the RF model has improved accuracy with FSA data, which can enhance the performance of the model and thus supports our objective that feature selection algorithms play a vital role in improving ML algorithms.

**Figure 5:**
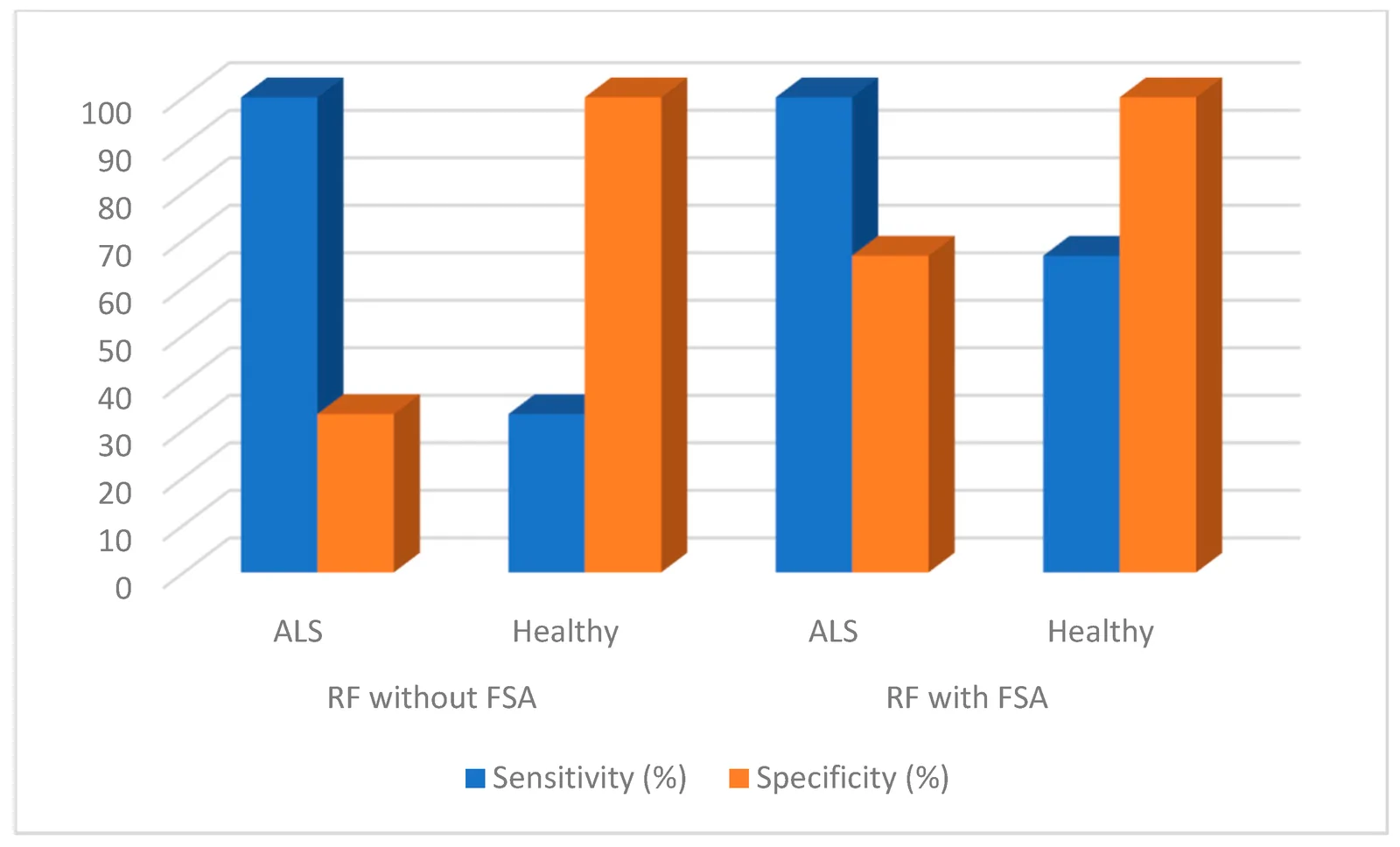
Comparison of sensitivity and specificity without and with FSA.

The sensitivity and specificity of our work, implemented by wrapper approaches, are enhanced from 33.33% to 66.67% for the healthy class and to almost 100% for ALS, as shown in Figure 5. The sensitivity shows that the model with FSA data has a better score than that without FSA data. The specificity shows an increase in the score due to the recursive feature elimination method. It has been observed that the RF-based ML model has the best scores with FSA data, describing the effectiveness of this model after FSA techniques are utilized.

**Figure 6:**
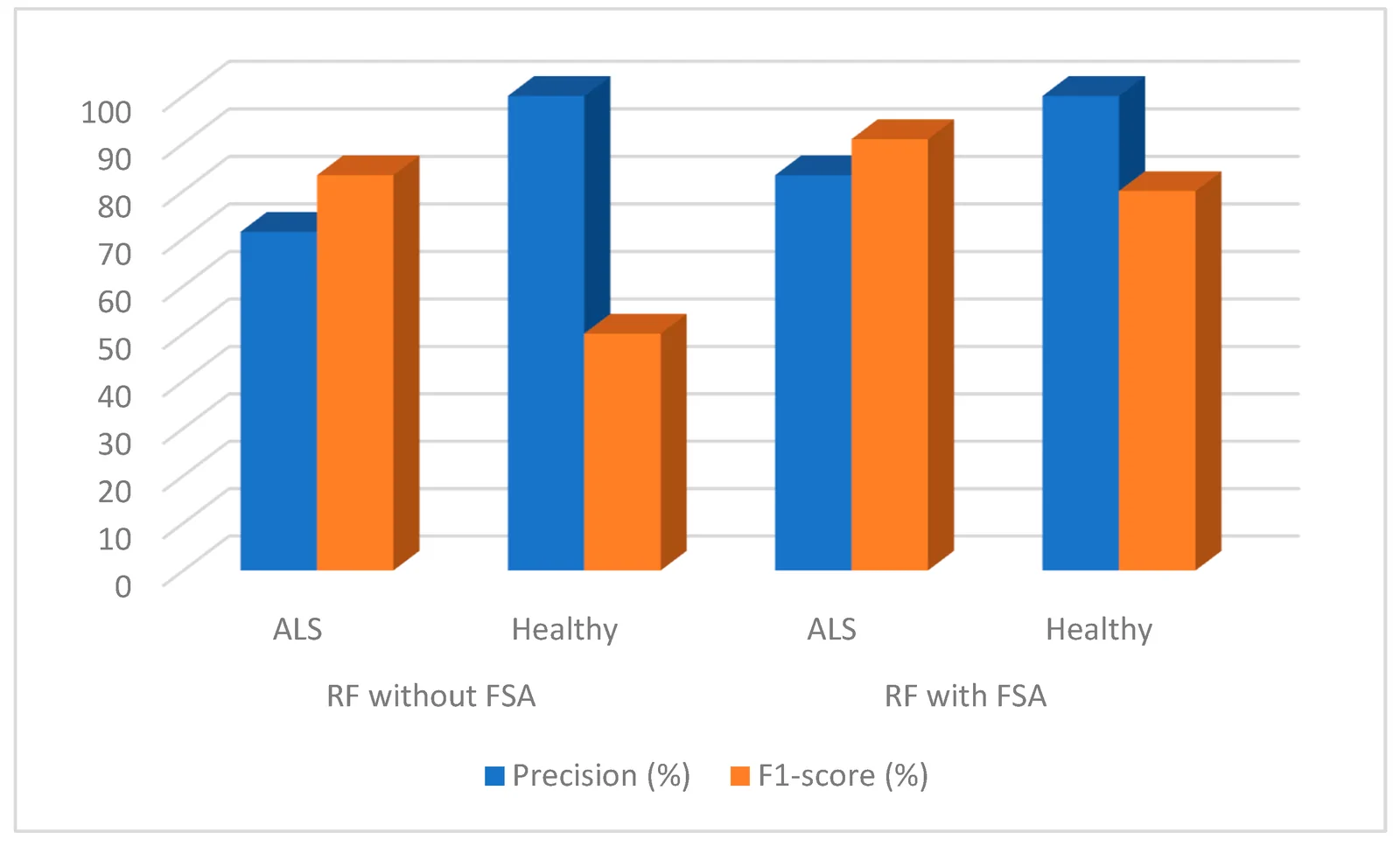
Comparison of precision and F1-score without and with FSA.

This strong performance demonstrates how well the model handles the difficulties associated with ALS prediction using EMG datasets. By eliminating extraneous features, the RF-based model not only attains exact correctness but also guarantees the model’s dependability. The precision increased somewhat from 71.43% to 83.33% in ALS and from 100% to 100% in healthy as a result of feature selection, while the F1-score rose significantly from 83.33% to 90.91% in ALS, and from 50% to 80% in the healthy class. This increase implies that the model was able to detect a higher percentage of genuine positive cases while keeping a relatively high precision, since feature selection improved the balance between precision and recall. The considerable rise in F1-score suggests an overall improvement in classification performance, while the slight drop in precision might be due to a slight increase in false-positive predictions. Figure 6 shows the combined graphical representation of precision and F1-score.

**Figure 7:**
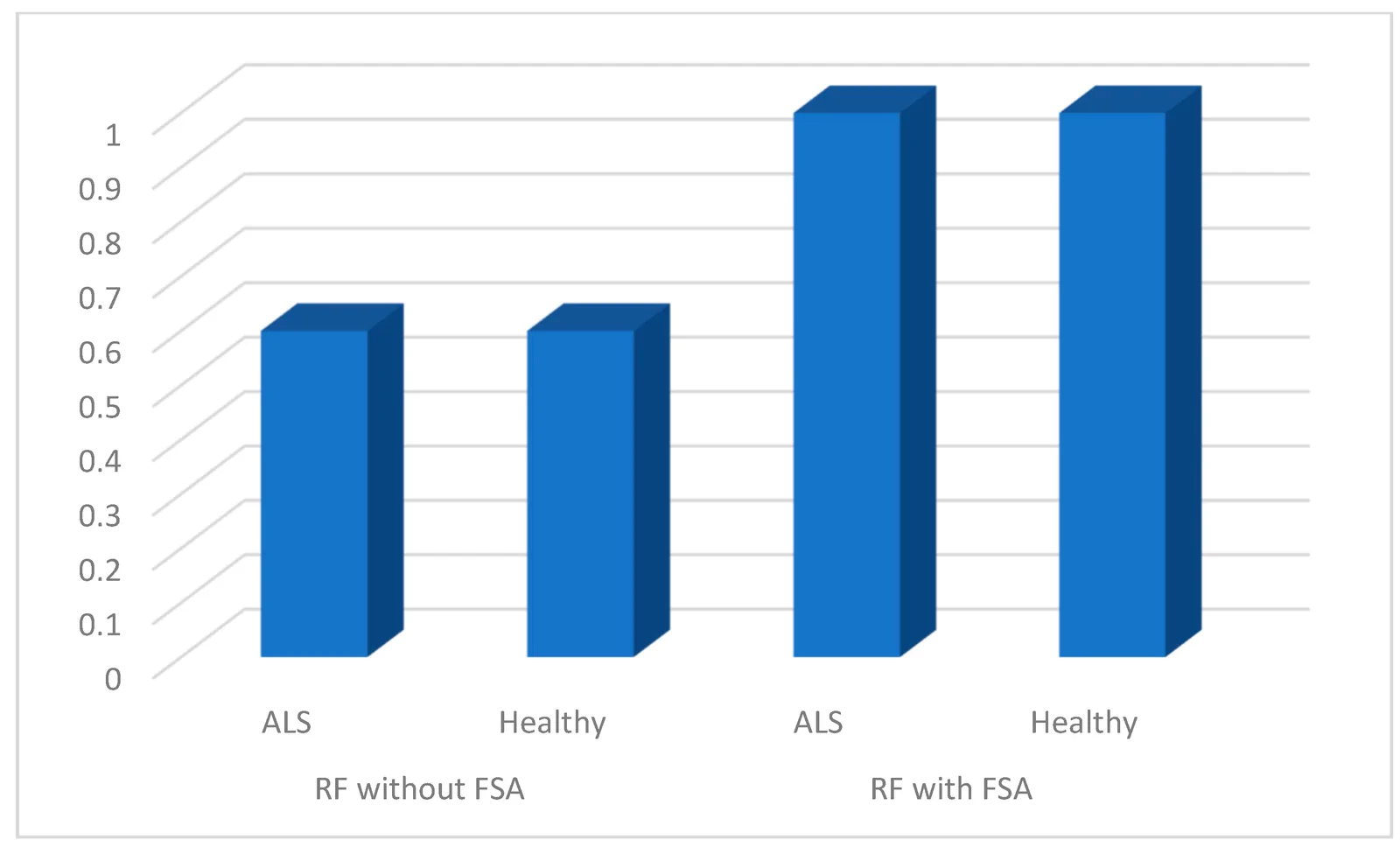
Comparison of AUC without and with FSA.

Figure 7 shows the graphical representation of the proposed model in terms of area under the curve (AUC). Here, the AUC with FSA data has better results and shows the effectiveness of the model; a higher AUC means better discrimination.

**Figure 8:**
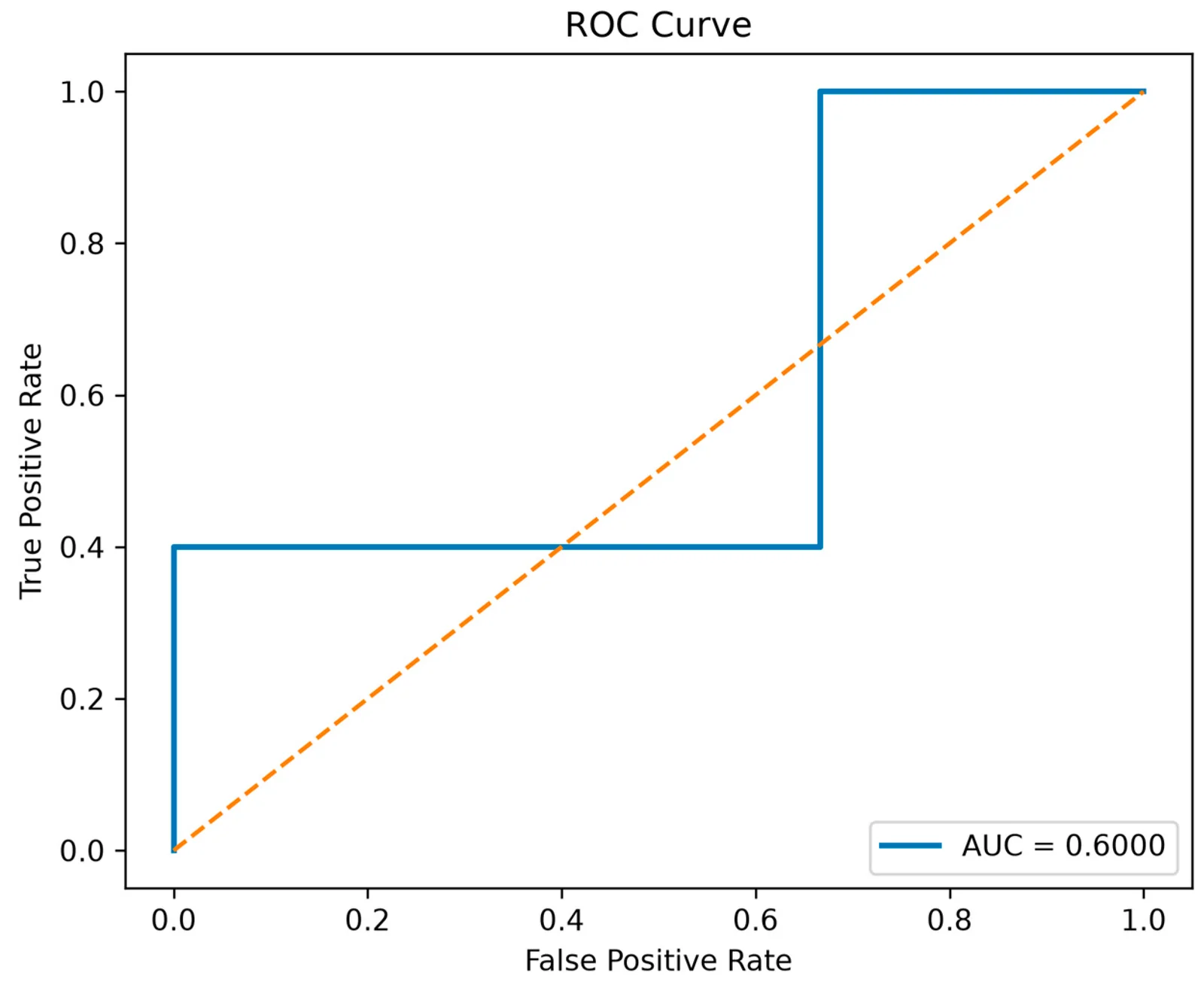
The ROC curve of the proposed Random Forest-based ML model without FSA data.

Furthermore, as shown in Figure 8, the suggested RF-based model performs noticeably better in AUC. Additionally. Figure 8 displays the receiver operating characteristic (ROC) curves for class ALS and class healthy for the suggested RF-based model, respectively. A classification model’s performance is shown graphically by the ROC curve. It draws attention to the trade-off between true positive rate (TPR) and false positive rate (FPR) in the ALS classification. An indicator of overall performance is provided by the area under the ROC curve (AUC).

**Figure 9:**
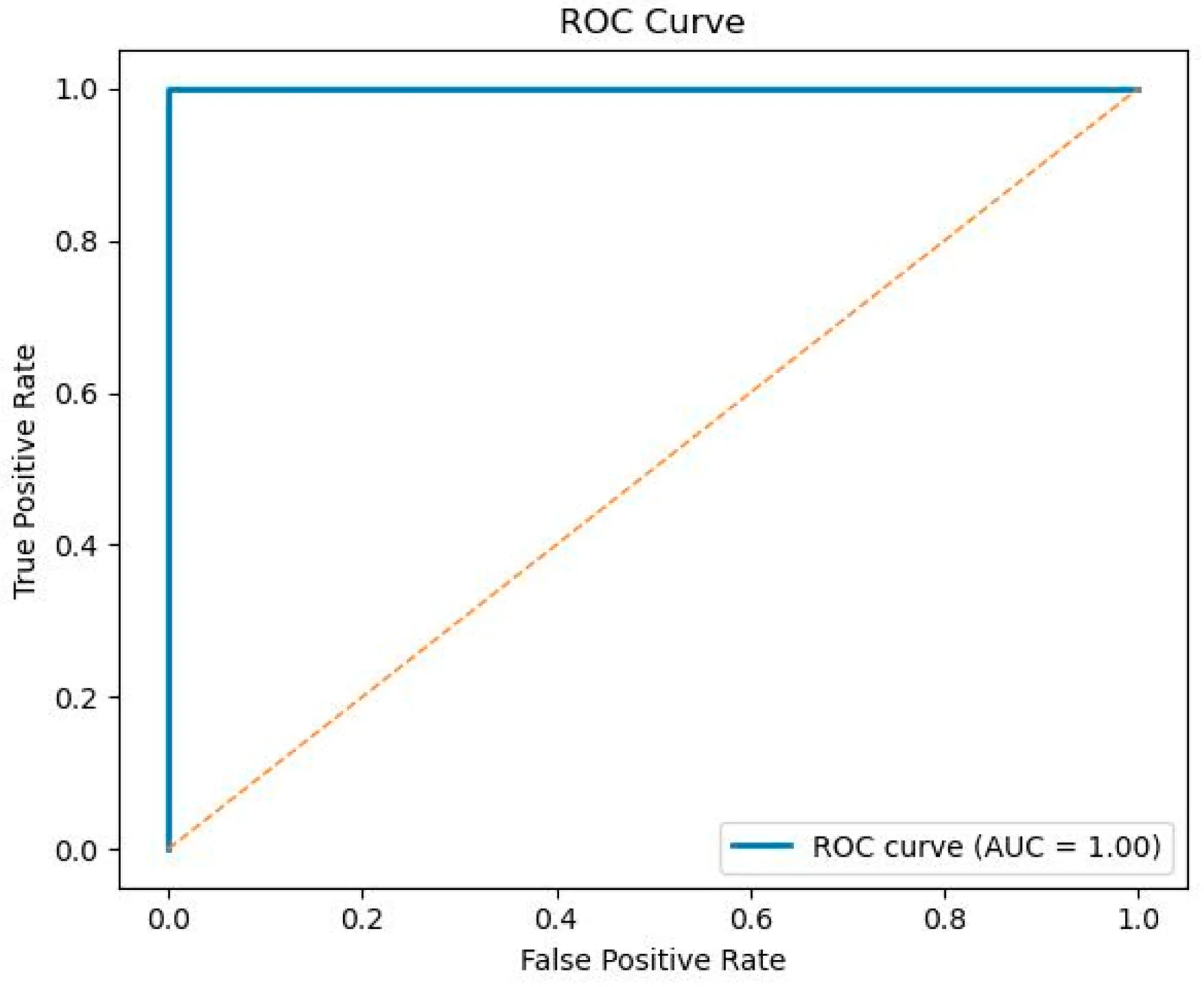
The ROC curve of the proposed Random Forest-based ML model with FSA data.

Near-perfect categorization is indicated by the ROC curves in Figure 9, which are in the upper-left corner. This shows that ALS and healthy are correctly identified using the RF-based prediction algorithm. The operational point of the model is shown by the blue and orange dots in Figure 9, which are located in the upper-left corner (AUC = 1.00). This suggests that the model avoids false positives, or incorrectly classifying healthy individuals, and accurately identifies all positive cases (prediction). Better performance is typically indicated by a greater AUC.

Our model predicts that positive sharp waves increase as fibrillation activity increases, which is clinically and physiologically plausible in EMG analysis. The majority of the points exhibit a steady upward trend, suggesting that the model predictions are fairly solid. A noteworthy high-value point could represent an anomaly or a severe case that the model predicted. The work suggests that, without FSA and with FSA data, the latter has better results and also suggests that EMG characteristics are connected and could co-occur in more severe ALS cases.

## 4. Discussion

To focus on how the feature selection algorithm (FSA) plays an important role in improving the ML performance, a comparative result was obtained on the performance of the RF-based model without FSA and with FSA data, as shown in Table 3 and Table 4, respectively. From the derived results, it is clearly seen how effective FSA can be and to what extent the with-FSA data can enhance the performance of the selected ML model. It is observed that the RF-based model was implemented without FSA data vs. with FSA data. The RF model showed an observed increase in accuracy from 75.00% without FSA to 87.50% with FSA, corresponding to an absolute increase of 12.5 percentage points. However, because the study included only 36 subjects, this difference should be interpreted cautiously. The observed increase represents a relatively small change in the number of correctly classified subjects and should not be considered evidence of statistically significant improvement without an appropriate paired statistical analysis. The results therefore indicate a potential benefit of FSA for RF-based ALS classification, but the observed improvement requires confirmation in larger and independently validated datasets. Our model has shown improvement in performance metrics with FSA, which has already been discussed in the results section.

Our research supports the idea that using machine learning techniques can significantly aid in the prediction of ALS using FSA data. The literature frequently ignores or scarcely discusses feature selection. However, we have demonstrated that the quality of a machine learning model can be greatly impacted by the use of an efficient feature selection technique. In fact, by determining which features had the biggest influence and applying an RF-based machine learning method, we were able to predict ALS with enhanced accuracy. Additionally, this strategy improves the interpretability of the classification and RF-based model by reducing the number of features from 16 to three in the ALS prediction. This study highlights how critical feature selection is to the modeling process and supports the notion that precise predictions may be made even with the scant data at our disposal. We were able to distinguish ALS patients from healthy individuals using an RF-based approach. Additionally, it is possible that characteristics not covered by our data, including particular comorbidity causes, unique genetic variations, or aspects of quality of life, could have a big impact on patients’ lives. Our findings support the feature selection algorithm (FSA), not earlier research [11,37,38] that used various machine learning techniques to determine the ALSFRS score’s slope. Over time, this suggested method improves forecasts and increases the model suitability for personalized predictive medicine. When paired with other methods, this approach can attenuate differences in a given subdomain, making it challenging to identify exact changes. This complexity is shown by ALS’s wide range of symptoms and development. This variation in how patients perceive and describe their symptoms may affect the outcome in the later stages of the illness, when patients may stabilize. The accuracy of ALS prediction may suffer greatly as a result of this heterogeneity and the metrics’ intrinsic limitations. In order to overcome these challenges, it is crucial to monitor ALS using objective biomarkers and additional measures like genetic characteristics [39,40] and imaging [41]. A multifaceted approach that incorporates different clinical evaluations and biological data would give a more comprehensive picture of the illness and its effects on patients’ motor function and quality of life. Additionally, this enhanced strategy would make it easier to use machine learning methods for forecasting. It should be mentioned that the peculiarities of our novel experimental EMG databases, particularly the lack of genetic data we use, make comparison with other comparable methods difficult. Furthermore, the quality of our model when applied to real-life data may be impacted by the fact that new EMG data are based on a particular demographic. Our suggested model robustness and prediction quality might be greatly improved by adding real-world data, which would also lessen the existing bias. However, our approach is different in that it uses the feature selection algorithm (FSA), which the latest prediction models do not include. Furthermore, whereas some studies [13,37,42] used feature selection, our study uses a wrapper-based approach. In our work, we have used a wrapper method, which identifies the optimal feature subsets that increase classification accuracy; wrapper approaches use a predetermined supervised or unsupervised learning algorithm. This approach frequently uses methods like recursive feature elimination (RFE) [27,28,29,30]. This method has the potential to significantly improve our understanding of ALS classification using EMG features, which would benefit the medical community. Wrapper methods allow for the fast selection of pertinent and ideal features while guaranteeing that physicians can conveniently access them for patient assessment. The choice of fibrillation potentials, positive sharp waves, and fasciculations is clinically expressive because these EMG characteristics represent abnormalities associated with motor-unit dysfunction and denervation. Their selection by the feature-selection procedure suggests that these characteristics contained useful discriminatory information for distinguishing ALS from healthy subjects within the present dataset. The increase in accuracy and F1-score after feature selection may indicate that removal of less informative features reduced unnecessary model complexity and allowed the classifier to focus on more discriminatory characteristics. However, the simultaneous change in specificity and AUC demonstrates that performance should not be interpreted using accuracy alone. Given the small sample size, relatively modest changes in individual observations can substantially influence performance estimates. Therefore, the observed improvement should be considered encouraging but preliminary and should be confirmed using larger independent datasets. It is also crucial to note that our strategy is not specific to ALS. Because of its adaptability, it can be used for a variety of illnesses, medical conditions, and prediction concerns. Our approach can therefore significantly contribute to the development of medical knowledge and practice, promoting research and innovation outside of the particular setting of ALS. The developed model is analyzed by comparing the datasets with and without FSA data. Both datasets were processed using FSA. Our model is the best choice for highlighting the prominent features, i.e., fibrillation, positive sharp waves, and fasciculation, which are effective for ALS prediction.

### Limitations

The present study has several limitations that should be considered when interpreting the findings. First, the relatively small sample size of 36 subjects limits the statistical power and generalizability of the proposed model. Although the RF model demonstrated an observed increase in accuracy from 75.00% without feature selection (FSA) to 87.50% with FSA, corresponding to an absolute increase of 12.5 percentage points, this improvement should not be interpreted as statistically significant based on accuracy alone. Future studies involving larger and independent cohorts should provide confidence intervals and perform appropriate paired statistical comparisons, such as McNemar’s test, where applicable.

Second, the present study used a cross-sectional design involving patients with clinically established ALS and healthy individuals. Although the proposed RF model demonstrated the ability to distinguish ALS from healthy participants using EMG-derived features, the study design does not establish prospective early prediction of ALS or prediction of disease onset. Longitudinal follow-up, information regarding the interval between symptom onset and electrophysiological assessment, and a cohort evaluated before definitive diagnosis were not available. Therefore, the findings should be interpreted as evidence of ALS classification/detection rather than confirmation of early prediction. Future prospective longitudinal studies involving participants at different stages of ALS and individuals with suspected disease are required to determine whether the identified EMG Features can support early-stage ALS detection.

Third, some EMG features were unavailable for certain participants, and participants with missing values in the required features were excluded from the final analysis. This complete-case approach may have reduced the effective sample size and could introduce selection bias if the missingness was not completely random. Future studies should evaluate larger datasets using appropriate missing-data handling strategies to assess the robustness and generalizability of the findings. A further limitation concerns the potential for overfitting associated with wrapper-based feature selection. Wrapper methods evaluate candidate feature subsets using a learning algorithm and may therefore identify a subset that performs particularly well on a small development dataset but does not generalize equally well to independent populations. Consequently, the selected features and observed performance should be considered preliminary rather than definitive evidence of generalizability.

Finally, although the proposed methodology was developed for ALS classification, the underlying feature selection and machine-learning framework may have potential applicability to other medical classification problems. However, such generalizability has not been demonstrated in the present study. Therefore, its applicability to other diseases or medical conditions should be investigated and validated using disease-specific datasets and independent cohorts rather than being assumed from the present results.

## 5. Conclusions and Future Work

The findings of this study indicate that fibrillation, positive sharp waves, and fasciculations were the most relevant features for distinguishing ALS from healthy individuals using the proposed Random Forest (RF)-based machine learning approach. The feature selection approach reduced the initial feature set from 16 to three features and was associated with an observed improvement in classification accuracy from 75.00% to 87.50%, corresponding to an absolute increase of 12.5 percentage points. These findings suggest that selected EMG features can provide useful information for machine-learning-based ALS classification. However, given the small sample size and cross-sectional study design, the results should be considered preliminary. The present study demonstrates ALS classification rather than prospective prediction of disease onset or definitive early ALS prediction. Therefore, further validation using larger, independent, and clinically diverse datasets is required to establish the robustness and generalizability of the proposed approach.

Future work will focus on validating the proposed RF-based classification framework using larger and independent cohorts to improve its robustness and generalizability. Prospective and longitudinal studies involving participants at different stages of ALS and individuals with suspected disease may help determine whether the identified EMG features can support early-stage ALS detection. Appropriate strategies for addressing class imbalance and missing data will also be investigated. In addition, subject-level validation and appropriate statistical analyses will be incorporated to provide more reliable estimates of model performance. Image-based representations of EMG signals may also be explored as an alternative input modality. Further research will investigate additional high-performing EMG features and other machine learning approaches to improve classification performance and assess the potential for real-time clinical applications. These developments may help establish a more reliable and clinically applicable machine-learning framework for ALS classification. Future studies will evaluate the selected EMG feature subset across multiple machine-learning classifiers under identical validation protocols to determine the generalizability and classifier dependence of the feature-selection effect.

## Figures and Tables

**Table 1:** Description and coding of the 16 input features used in the initial dataset.

S. No.	Feature	Definition	Coding
1	Side	Indicates the anatomical side from which the EMG examination was performed.	Categorical: Right = 0, Left = 1
2	Age	Age of the participant at the time of EMG examination.	Continuous; measured in years
3	Sex	Biological sex of the participant.	Categorical: Male = 0, Female = 1
4	Insertional Activity	Electrical activity observed when the needle electrode is inserted or moved within the muscle. Abnormal/prolonged insertional activity may indicate muscle membrane instability or denervation.	Categorical: 0 = Yes, 1 = No
5	Fibrillation (Fibs.)	Spontaneous electrical discharges from individual muscle fibers, commonly associated with active denervation.	Categorical: 0 = Absent, 1 = Present
6	Positive Sharp Waves (PSW)	Spontaneous EMG discharges characterized by an initial positive deflection followed by a slower negative component; commonly associated with denervation.	Categorical: 0 = Absent, 1 = Present
7	Fasciculations (Fasc.)	Spontaneous discharges produced by the involuntary activation of individual motor units.	Categorical: 0 = Absent, 1 = Present
8	Other Spontaneous Discharge (S.D.)	Records spontaneous EMG activity other than fibrillation potentials, positive sharp waves, and fasciculations.	Categorical: 0 = Yes, 1 = No
9	Recruitment	Describes the number and activation pattern of motor units recruited during voluntary muscle contraction.	Categorical: 0 = Yes, 1 = No
10	Normal Motor Unit Action Potential (MUAP)	Represents the characteristics of normally functioning motor unit action potentials during voluntary muscle activation.	Categorical: 0 = Absent, 1 = Present
11	Polyphasia MUAP	Indicates the presence/proportion of polyphasic motor unit action potentials, which have an increased number of waveform phases.	Categorical: 0 = Absent, 1 = Present
12	Amplitude	The voltage magnitude of the recorded motor unit action potential.	Continuous; measured in mV or μV
13	Duration	The time interval over which an individual MUAP waveform persists.	Continuous; measured in milliseconds (ms)
14	Interference Pattern (Intf. Patt.)	Represents the combined electrical activity of multiple motor units during strong voluntary muscle contraction.	Categorical: 0 = Yes, 1 = No
15	Neurogenic Pattern	Indicates an EMG pattern suggestive of neurogenic motor-unit changes, such as reduced recruitment and changes in MUAP morphology.	Categorical: 0 = Yes, 1 = No
16	EMG Impression	Overall interpretation of the EMG examination based on the observed electrophysiological findings.	Categorical: 0 = Yes, 1 = No

**Table 2:** Clinical and demographic information of ALS patients and healthy persons (*n* = 21 for ALS and *n* = 15 for healthy persons).

ALS (*n* = 21)	Mean ± SD	Range	Healthy (*n* = 15)
Age (years)	44.95 ± 10.8	24–58	22–51
Disease duration (Years)	2 ± 0.816	1–3	-
ALS-FRS-R Score (48)	29.24 ± 10.33	10–48	-
mRS Score (6)	2.1 ± 1.70	0–5	-
Gait Score (30)	17.62 ± 9.26	0–30	-
MOCA Score (30)	23.33 ± 7.15	0–30	-
MMSE Score (30)	23.24 ± 7.11	0–30	-

**Table 3:** Proposed model findings with different performance metrics without FSA for both classes.

Class	Sensitivity (%)	Specificity (%)	Accuracy (%)	Precision (%)	F1-Score (%)	AUC
ALS	100	33.33	75	71.43	83.33	0.600
Healthy	33.33	100	75	100	50	0.600

**Table 4:** Proposed model findings with different performance metrics and FSA for both classes.

Class	Sensitivity (%)	Specificity (%)	Accuracy (%)	Precision (%)	F1-Score (%)	AUC
ALS	100	66.67	87.50	83.33	90.91	1
Healthy	66.67	100	87.50	100	80	1

**Table 5:** Earlier work with different features used in ALS disease using statistical analysis.

Model/Year	Dataset	Number of Features Used	Number of Healthy Person	Number of ALS Patients	Statistical Analysis Perform	*p*-Value
Van der Heijden, A. et al., 1994 [7]	EMG	1 (fasc.)	60	-	Mann–Whitney U-test.	<0.001
de Carvalho et al., 2008 [8]	EMG	3 (Fibs, fasc., PSW)	-	68	-	-
Sleutjes, B. T. et al., 2016 [34]	EMG	1 (fasc.)	10	30	Mann–Whitney U-test.	<0.001
Jingwen Liu et al., 2021 [35]	Muscle ultrasonography	1 (fasc.)	54	88	Mann–Whitney U-test.	<0.05
Yao, Y. et al., 2024 [36]	Genetic	1 (Fibs.)	-	138,086	Mendelian randomization	=0.035

## Data Availability

Due to ethical or privacy concerns, the datasets created and/or analyzed for this research are not publicly accessible. However, upon reasonable request, the data can be obtained from the corresponding author.
